# Wildfire smoke exposure and emergency department visits in Washington State

**DOI:** 10.1088/2752-5309/acd3a1

**Published:** 2023-05-25

**Authors:** Annie Doubleday, Lianne Sheppard, Elena Austin, Tania Busch Isaksen

**Affiliations:** 1 Department of Environmental and Occupational Health Sciences, University of Washington, Seattle, WA, United States of America; 2 Department of Biostatistics, University of Washington, Seattle, WA, United States of America

**Keywords:** wildfire smoke, climate change, risk communication, syndromic surveillance

## Abstract

Wildfires are increasing in prevalence in western North America due to changing climate conditions. A growing number of studies examine the impact of wildfire smoke on morbidity; however, few evaluate these impacts using syndromic surveillance data that cover many emergency departments (EDs). We used syndromic surveillance data to explore the effect of wildfire smoke exposure on all-cause respiratory and cardiovascular ED visits in Washington state. Using a time-stratified case crossover design, we observed an increased odds of asthma visits immediately after and in all five days following initial exposure (lag 0 OR: 1.13; 95% CI: 1.10, 1.17; lag 1–5 ORs all 1.05 or greater with a lower CI of 1.02 or higher), and an increased odds of respiratory visits in all five days following initial exposure (lag 1 OR: 1.02; 95% CI: 1.00, 1.03; lag 2–5 ORs and lower CIs were all at least as large) comparing wildfire smoke to non-wildfire smoke days. We observed mixed results for cardiovascular visits, with evidence of increased odds emerging only several days following initial exposure. We also found increased odds across all visit categories for a 10 *μ*g m^−3^ increase in smoke-impacted PM_2.5_. In stratified analyses, we observed elevated odds for respiratory visits among ages 19–64, for asthma visits among ages 5–64, and mixed risk estimates for cardiovascular visits by age group. This study provides evidence of an increased risk of respiratory ED visits immediately following initial wildfire smoke exposure, and increased risk of cardiovascular ED visits several days following initial exposure. These increased risks are seen particularly among children and younger to middle-aged adults.

## 1. Background

Wildfires are increasing in prevalence in western North America due to changing climate conditions, including prolonged droughts and heat waves [1]; changing land-use resulting in an increase in the wildland-urban interface [2]; and decades of poor forest management [3, 4]. Accordingly, smoke from wildfires continues to impact large populations over prolonged time periods in western North America, resulting in worsening air quality over the past decade, reversing improvements in air quality overall [5–7]. Recent toxicology and epidemiology studies suggest increased toxicity comparing wildfire smoke-influenced PM_2.5_ to PM_2.5_ without wildfire smoke [8–10]. Thus, characterizing the health impacts of wildfire smoke is critical in a region’s efforts to mitigate adverse health outcomes from smoke exposure.

Studies examining the impact of wildfire smoke exposure on morbidity are increasingly abundant [11–21], with several hospitalization and emergency department (ED) visit studies. These studies consistently estimate increased risk of respiratory hospitalizations and ED visits associated with wildfire smoke exposure [11–15, 17–19]. However, the results for cardiovascular hospitalizations and ED visits are inconsistent across studies, including a decreased risk, no change in risk, and an increase in risk [11–13, 15–19].

To our knowledge, there are only a few studies utilizing syndromic surveillance data in a wildfire smoke context [17, 20–22]. Syndromic surveillance is a near real-time monitoring system that requires all EDs in Washington to report healthcare encounter visit data to help rapidly identify outbreaks and other public health emergencies [23, 24]. Utilization of syndromic surveillance data increases our power to detect an effect due to the high frequency of ED visits compared to more severe outcomes such as hospitalization or mortality. Further, to date, there are only two statewide wildfire smoke epidemiologic studies in Washington, and only one examining non-mortality outcomes [12, 25]. Gan *et al* examined hospitalization outcomes during summer 2012 and found an increased risk of respiratory hospitalizations associated with wildfire smoke, as characterized by three different exposure metrics. However, due to lack of comprehensive data sources and difficulty accessing health data, no studies have examined the effect of wildfire smoke exposure across several years of morbidity data in Washington. We examined both the overall impact and the intensity of wildfire smoke exposure on ED visits in Washington during the summers of 2017–2020 using a wide-ranging dataset of EDs covering all of Washington. Our results further elucidate our understanding of how the presence and intensity of wildfire smoke impacts ED visits. Our results will help inform Washington state and local agency efforts to craft more targeted risk communication and interventions.

## 2. Methods

### 2.1. Study location and time period

The study period spanned June-September, 2017–2020, and included all reporting EDs in Washington state. We chose June–September as all major wildfire smoke events across this time period occurred during these months.

### 2.2. Exposure assessment

We used two primary exposure metrics: (1) a binary indicator of wildfire smoke-impacted days, and (2) wildfire smoke-impacted PM_2.5_ concentration at the zip code level. The binary indicator of wildfire smoke gives results that are easier to communicate to the public and to practitioners, and the smoke-impacted PM_2.5_ concentration increases our understanding of how the intensity of exposure impacts health.

To create the binary metric, we assigned wildfire smoke days based on a combination of modeled and monitored PM_2.5_ [25]. Briefly, 4 × 4 km grid cells from the *Air Indicator Report for Public Awareness and Community Tracking (AIRPACT-4)* model domain in Washington were assigned the daily average monitored concentration from the nearest regulatory air quality monitor, based on agreement between the summertime average at the grid cell (based on the AIRPACT modeled concentration, scaled by the monitored concentration) and the summertime average at the nearest monitor. See Note S1 in the supplementary material for details. We then computed the daily population-weighted average zip code-level PM_2.5_ concentration to match the spatial resolution of the ED visit data.

We identified wildfire smoke days through an iterative process, described elsewhere [25]. Briefly, we tried a threshold of 15 *μ*g m^−3^, corresponding to the 99th percentile of measured PM_2.5_ concentrations across two years with minimal smoke impacts. However, most days above this threshold in urban areas were not wildfire smoke impacted, while some days below this threshold in rural areas were wildfire smoke impacted. We thus applied a more nuanced approach to better capture wildfire smoke days in both urban and rural areas. For the binary analysis, smoke days are defined as:

(a) All zip code-days greater than 20.4 *μ*g m^−3^.
(b) Zip code-days with a 24 h average PM_2.5_ concentration between 9 and 20.4 *μ*g m^−3^ must meet the following set of criteria to be considered a smoke day [25]:
  1. the day must be part of an event in which at least 2 of 3 consecutive days are greater than 9 *μ*g m^−3^;
  2. one of the days in the 3 day window must be greater than 15 *μ*g m^−3^; and
  3. for urban areas (Seattle, Tacoma, Spokane), at least 50% of the air monitors in those areas must be greater than 9 *μ*g m^−3^.

We chose the 20.4 *μ*g m^−3^ based on the cut point between Moderate and Unhealthy for Sensitive Groups in the previously used Washington Air Quality Advisory. The lower cut point of 9 *μ*g m^−3^ was used after sensitivity analyses that found wildfire smoke impacts at low levels, corresponding to 9 *μ*g m^−3^.

We used this exposure grid to calculate wildfire smoke PM_2.5_ concentration for each zip code-day. Wildfire smoke PM_2.5_ was defined as the total PM_2.5_ on smoke-impacted days (using the definition above) minus typical PM_2.5_, where typical PM_2.5_ was defined as the median PM_2.5_ concentration for each zip code and month on non-smoke impacted days. All non-smoke days were assigned a smoke PM_2.5_ concentration of 0 *μ*g m^−3^. By subtracting out typical PM_2.5_ levels by season (month) and location (zip code), this focuses our analysis on smoke-impacted PM_2.5_ using an approach similar to that used in other wildfire smoke-health studies [26, 27]. We used this wildfire smoke-impacted PM_2.5_ for our primary analysis examining the impact of wildfire smoke intensity on ED visits.

In a sensitivity analysis, we re-ran our primary analysis using the National Oceanic and Atmospheric Administration’s (NOAA) daily hazard mapping system (HMS) smoke plumes to determine the binary exposure metric [28]. Briefly, trained NOAA analysts created smoke polygons over North America based on satellite imagery from GOES-16 and GOES-17. The polygons were designated as light, medium, or heavy smoke. We used the polygons categorized as ‘Heavy’ smoke, as these are more likely to be at ground level, reducing misclassification. If a smoke plume intersected with the zip code on a specific day, we categorized that zip code as smoke impacted [16]. We considered using smoke plumes categorized as ‘Medium’, but found they introduce many false positives.

### 2.3. Outcome assessment

We used selected ED visits from Washington state’s syndromic surveillance system (Rapid Health Information NetwOrk (RHINO)) for our study period. The system contains patient demographic information, data about the visit, and clinical information pertinent to the visit. We examined the following groups of diagnoses as our outcomes of interest, using all listed diagnosis codes for each visit: all non-traumatic (ICD-10-CM codes: A01-R99), all-cause respiratory (ICD-10-CM codes: J01-J99), asthma (ICD-10-CM codes: J45), all-cause cardiovascular (including cerebrovascular outcomes) (ICD-10-CM codes: I05-I52, I60-63, I65-69, G45), and myocardial infarction (MI) (ICD-CM-10: I21). We additionally queried the chief complaint text field to ensure we captured all ED visits in each outcome category (described in table S1 in the supplemental material). We leveraged queries developed by the Washington state Department of Health RHINO [23] team to maintain consistency and utilize queries that have been previously developed and tested.

### 2.4. Inferential analysis

We leveraged a time-stratified case-crossover design to evaluate the effect of wildfire smoke presence versus absence and wildfire smoke intensity on ED visits. This design uses conditional logistic regression to compare exposures within individuals during a time-stratified referent window. The exposure on the index date, the day of the ED visit or a prior day for lagged exposures, is compared to the exposure on referent days, defined as the same day of the week and month of the index day. We report the odds ratio (OR) on wildfire smoke days compared to non-wildfire smoke days using the daily patient zip-code level binary smoke indicator, described above. We report the OR separately for lag days 0–5 for respiratory outcomes, or lag days 0–7 for cardiovascular outcomes. We chose *a priori* to extend the lag period to 7 d for the cardiovascular outcomes based on evidence suggesting an increase in cardiovascular-related mortality several days following initial exposure [21, 27]. Lag 0 represents initial exposure on the day of the ED visit, lag 1 represents initial exposure on the day prior to the visit, and so on. We adjusted for time- and zip code-specific humidex at lag 0 [29], a measure of temperature and humidity, modeled as a natural cubic spline with three degrees of freedom, as practiced elsewhere [30, 31] (see equation S1 in the supplemental material for the model notation).

The syndromic surveillance system was actively onboarding EDs in Washington during the study period. Thus, we ran the binary conditional logistic regression model separately for each year and combined the results using a fixed effect meta-analysis to yield an overall OR for the study period [32]. This method upweights years with smaller standard errors (more data). While we do not expect year-specific results to be homogenous, Rice *et al* show that the overall effect estimated from a fixed effect meta-analysis is unbiased if the individual study effects are unbiased [32]. We report results using a mixed effects meta-analysis as a sensitivity analysis.

In a secondary analysis, we reported results for all-cause respiratory, asthma, all-cause cardiovascular, and MI visits stratified by age group (0–4, 5–18, 19–44, 45–64, 65–84, ⩾85), using the same model form as the binary analysis.

To understand how the intensity of wildfire smoke impacts ED visits, we again used the time-stratified case-crossover design with conditional logistic regression. We used the continuous wildfire smoke PM_2.5_ exposure metric over the pre-defined lag period using a distributed lag model. A distributed lag model uses an assumed lag structure model to distribute the effect of the exposure across the entire lag period rather than on one lag day, as done in a single lag model [33]. The resulting effect estimates describe the lag-specific and cumulative impact of a 10 *μ*g m^−3^ increase in wildfire smoke PM_2.5_ across the lag period, taking into account any smoke exposure during the lag period for the cumulative OR [22, 27, 30, 34]. Estimates from a distributed lag model cannot be directly compared to results from a single exposure day model. We used the `dlnm` R package [33], and modeled the exposure-outcome relationship linearly, with the lag modeled as a natural cubic spline with three degrees of freedom, as documented in similar studies [22, 27].

We additionally sought to visualize the wildfire smoke PM_2.5_-health effect relationship within the context of our existing public health messaging. We used the same model structure as in the intensity analysis, using a total PM_2.5_. We modeled total PM_2.5_ using a natural cubic spline with one knot at 55 *μ*g m^−3^, corresponding to the US Environmental Protection Agency’s air quality index (AQI) cut point between ‘Unhealthy for Sensitive Groups’ and ‘Unhealthy’ for PM_2.5_. We estimated the dose response curve for a 10 *μ*g m^−3^ in total PM_2.5_ for respiratory visits on lag day 1 plotting the curve over the AQI categories for visualization.

We ran several sensitivity analyses. First, we evaluated the binary model with and without COVID-19 diagnoses at time of treatment (table S1). This only impacts data from 2020. We then re-ran the binary model excluding all cases from 2017, and then separately by year due to differences in coverage and patterns among reported cases by year (table 1). We also re-evaluated the binary model results using a mixed effect meta-analysis rather than a fixed effect meta-analysis to allow the effects to differ by year. Next, we re-ran the binary model assigning smoke presence versus absence using NOAA’s HMS smoke plumes, as described above. Finally, we ran all models using a quasi-Poisson time series analysis, adjusting for humidex, day of the week, month, year, and a year-specific log zip code-level population offset. Unlike the conditional logistic regression models used in our primary analyses, this model does not make assumptions about patient readmissions during the referent period.

**Table 1.** Summary of emergency department visit characteristics.

Characteristic (N (%))	All years	2017	2018	2019	2020
Total study visits (all non-traumatic)^a^	1864 470	235 314	424 833	629 702	574 621
EDs ever reporting	94 (100)	39 (41)	66 (70)	83 (88)	94 (100)
Average study visits per day	3821	1929	3482	5161	4710
Age group					
0–4	82 040 (4)	13 389 (6)	21 028 (5)	30 728 (5)	16 895 (3)
5–18	139 878 (8)	20 559 (9)	32 700 (8)	49 459 (8)	37 160 (7)
19–44	692 257 (37)	86 855 (37)	155 699 (37)	228 832 (36)	220 871 (38)
45–64	483 231 (26)	61 352 (26)	110 194 (26)	160 654 (26)	151 031 (26)
65–84	371 043 (20)	42 211 (18)	83 644 (20)	125 789 (20)	119 399 (21)
⩾85	94 196 (5)	10 892 (5)	21 449 (5)	32 673 (5)	29 182 (5)
Missing	1825 (<1)	56 (<1)	119 (<1)	1567 (<1)	83 (<1)
Race					
American Indian/ Alaska Native	37 356 (2)	4263 (2)	8705 (2)	11 700 (2)	12 688 (2)
Asian	61 084 (3)	4304 (2)	12 854 (3)	23 731 (4)	20 195 (4)
Black	138 226 (7)	16 023 (7)	30 145 (7)	49 321 (8)	42 737 (7)
Native Hawaiian/ Other Pacific Islander	21 456 (1)	637 (<1)	3593 (1)	9328 (2)	7898 (1)
White	1363 223 (73)	172 581 (73)	323 787 (76)	451 353 (72)	415 502 (72)
Other	142 720 (8)	3570 (2)	24 266 (6)	60 276 (10)	54 608 (10)
Not reported	100 405 (5)	33 936 (14)	21 483 (5)	23 993 (4)	20 993 (4)
Ethnicity					
Hispanic	182 277 (10)	21 635 (9)	38 544 (9)	63 102 (10)	58 996 (10)
Not Hispanic	1587 564 (85)	176 907 (75)	367 681 (87)	545 404 (87)	497 572 (87)
Not reported	94 629 (5)	36 772 (16)	18 608 (4)	21 196 (3)	18 053 (3)
Sex					
Female	1016 769 (55)	126 723 (54)	232 670 (55)	345 510 (55)	311 866 (54)
Male	847 539 (45)	108 584 (46)	192 145 (45)	284 138 (45)	262 672 (46)
Outcome					
All Non-traumatic^a^	1864 470	235 314	424 833	629 702	574 621
All Respiratory	353 717 (19)	38 137 (16)	73 777 (17)	123 592 (20)	118 211 (21)
Asthma	68 293 (4)	4981 (2)	10 246 (2)	24 915 (4)	28 151 (5)
All Cardio	537 007 (29)	46 565 (20)	99 144 (23)	195 298 (31)	196 000 (34)
Myocardial infarction	131 668 (7)	13 645 (6)	29 987 (7)	45 679 (7)	42 357 (7)

*Note:* percentages are rounded to the nearest whole number and may not add to 100%.

^a^ All study visits are non-traumatic. All other visit categories are not mutually exclusive. A visit can be counted in multiple outcome categories, as all listed diagnosis codes are used to identify the outcome.

## 3. Results

The Washington syndromic surveillance platform began collecting ED visit data in 2011 and was still onboarding EDs during the study period, resulting in fewer visits in 2017 compared to 2019 and 2020 (table 1). All-cause respiratory visits make up 19% of all visits recorded in the dataset over the four summers, while all-cause cardiovascular visits make up nearly 29% of visits.

Table 2 shows average total PM_2.5_ concentration and humidex at the zip code level averaged across the study region by year, and by wildfire smoke day. The average concentration on non-smoke days is consistent across the study period, however the total PM_2.5_ concentration on smoke days varies by year. The 2020 wildfire smoke season had nearly three times higher average smoke day PM_2.5_ concentration than the next highest year, 2018. The 2019 wildfire smoke season experienced the lowest average smoke day PM_2.5_ concentration of the four years, and the fewest number of smoke days by zip code. Additionally, most (97%) smoke days were a part of a multi-day event, defined as at least two consecutive smoke days by zip code. The distribution of PM_2.5_ on smoke versus non-smoke days, a summary of exposure at the referent window level (each individual set of referent and index days), and a comparison of the primary exposure model and the HMS smoke plume exposure model are summarized in figure S1 and tables S2, S3.

**Table 2.** Summary of PM_2.5_ and humidex across the study period and by year.

		All years	2017	2018	2019	2020
PM_2.5_ (*μ*g m^−1^ ^c^), Mean (SD)						
Overall^a^		9.6 (3.6)	10.4 (4.4)	10.4 (5.2)	4.2 (1.1)	13.6 (4.4)
Non-smoke-days^b^		4.3 (1.1)	4.5 (1.3)	4.4 (1.0)	4.0 (1.0)	4.1 (0.9)
Smoke days		48.7 (9.8)	33.4 (8.8)	37.3 (9.2)	17.7 (3.9)	94.7 (32.0)
Number of smoke days		14.4 (12.1)	22.4 (11.2)	21.2 (12.4)	1.7 (3.5)	12.6 (4.3)
Index days^c^		9.4 (25.2)	10.1 (18.6)	9.7 (16.9)	4.3 (2.7)	14.4 (40.5)
Referent days		9.3 (24.8)	10.3 (18.5)	9.6 (16.7)	4.3 (2.7)	14.2 (39.8)
Humidex (degrees C), Mean (SD)						
Overall humidex		25.5 (2.3)	26.0 (2.4)	25.5 (2.2)	25.5 (2.1)	25.2 (2.4)
Non-smoke days		25.1 (2.2)	25.1 (2.2)	24.7 (2.0)	25.4 (2.0)	25.2 (2.5)
Smoke days		28.7 (2.3)	30.8 (2.4)	28.8 (2.6)	33.0 (2.4)	25.0 (1.8)
Index days		25.5 (5.5)	25.8 (6.4)	25.5 (5.6)	25.6 (5.1)	25.3 (5.3)
Referent days		25.5 (5.5)	25.8 (6.6)	25.4 (5.7)	25.6 (5.1)	25.3 (5.4)

^a^ All metrics are averages of zip code-level averages except summaries on index and referent days.

^b^ Smoke and non-smoke days defined by primary binary exposure definition at the patient’s zip code-day level.

^c^ Summaries of index and referent days summarize PM_2.5_ and humidex across the full case-crossover dataset which includes all referent windows.

**Figure 1.**
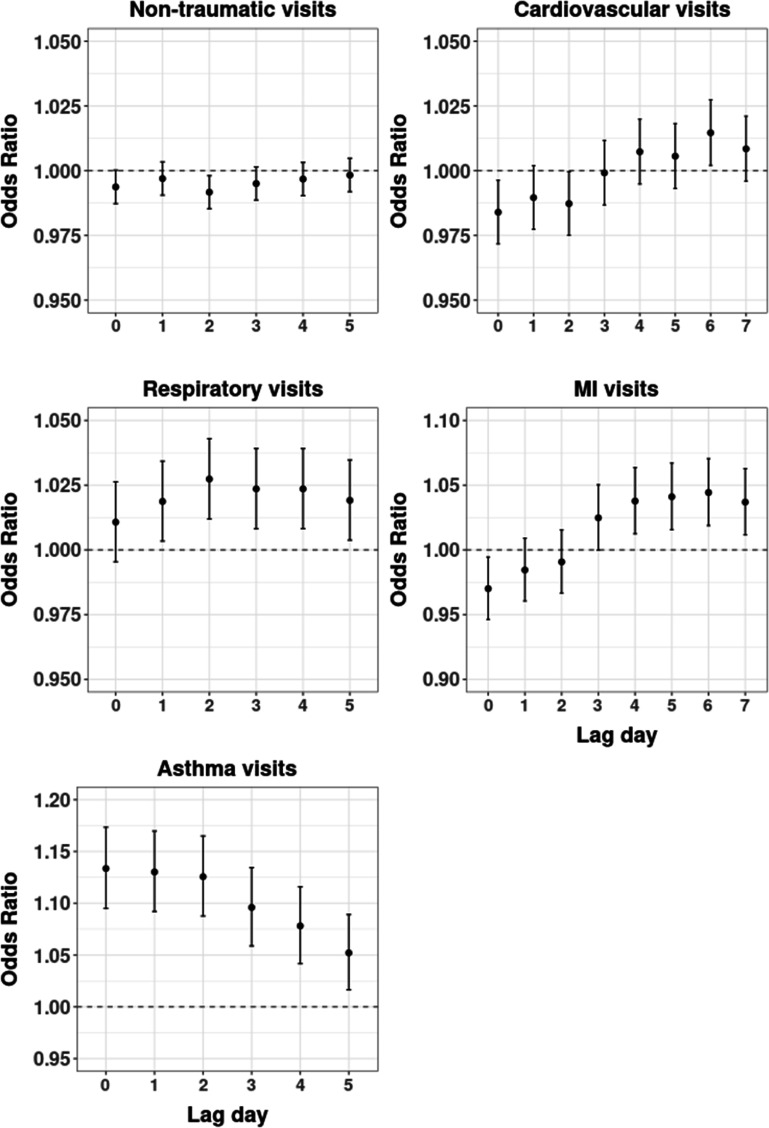
Lag-specific odds ratios of ED visits by outcome comparing wildfire smoke versus non-wildfire smoke days, controlling for humidex. Note *y*-axes vary by outcome to allow for visualization of results. All-cause respiratory includes asthma visits and all-cause cardiovascular includes MI visits. Visit categories are not mutually exclusive.

In our presence versus absence analysis, we examined the impact of initial wildfire smoke exposure on ED visits using single-day binary exposure models (figure 1; table S4). We observed both protective and null effects for all non-traumatic ED visits comparing smoke days to non-smoke days (figure 1). For all-cause respiratory ED visits, we observed no increase on lag 0, and an increase in odds across lags 1–5, with peak odds on lag 2 (OR: 1.03; 95% CI: 1.01, 1.04). For asthma-related ED visits, there was an increase in the odds of visits on smoke versus non-smoke days across all lags, with peak odds on the initial day of exposure (OR: 1.13, 95% CI: 1.10, 1.17). For all-cause cardiovascular visits, the single-day ORs were inconsistent across the lag period, including a decrease (lag 0 and lag 2), no increase (lag 1, 3–5, 7), and an increase (lag 6, OR: 1.02, 95% CI: 1.00, 1.03) in the odds of visits on smoke days compared to non-smoke days (table S4). Similarly, for MI visits, we observed a range of effects consistent with a decrease (lag 0), no increase (lag 1–3), and an increase (lag 4–7) in the odds, with peak odds on lag 6 (OR: 1.04, 95% CI: 1.02, 1.07). Results from a mixed effect meta-analysis show similar trends with slightly muted effects and wider confidence intervals (table S5).

**Figure 2.**
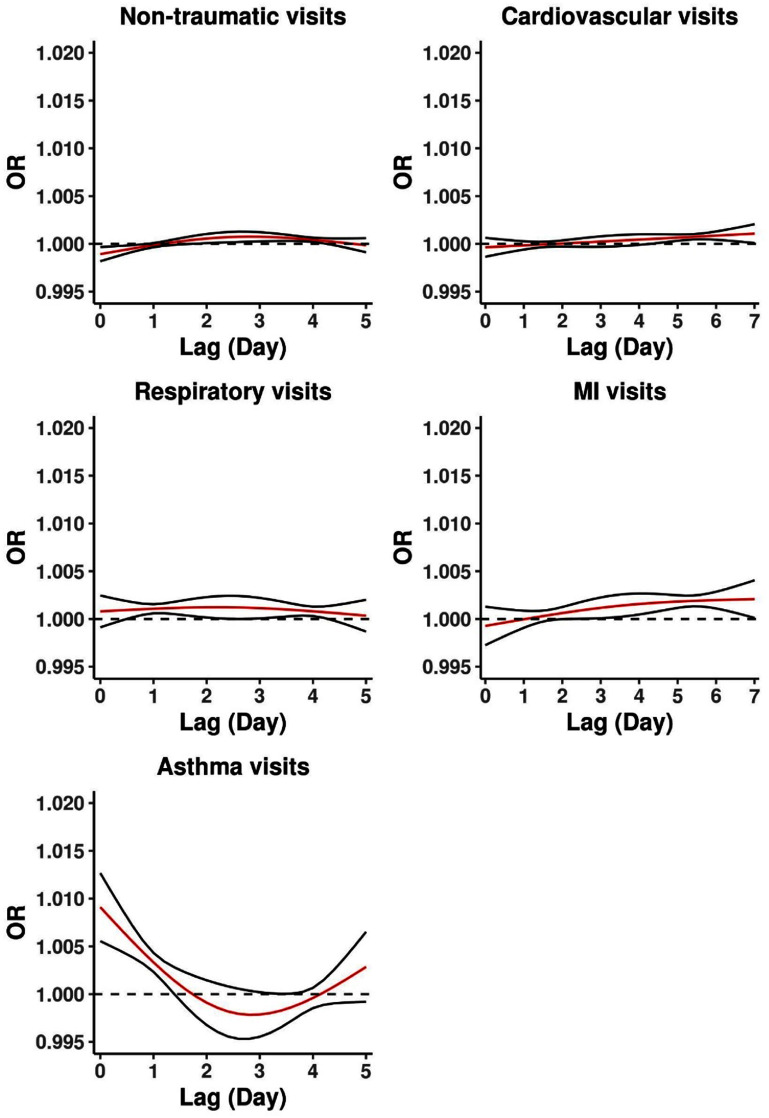
Lag-specific odds ratios of outcome-specific ED visits for a 10 *μ*g m^−3^ increase in wildfire smoke PM_2.5_. Exposure is modeled with a distributed lag model. ORs are plotted on the same scale for comparison across outcome groups.

In our second primary analysis, we assessed the intensity of wildfire smoke exposure on ED visits. We separately examined the lag-specific and cumulative effects of exposure across 6 and 8 d for respiratory and cardiovascular visits, respectively. For all non-traumatic visits, we observed a lag-specific OR of about 1.00 on each lag day per 10 *μ*g m^−3^ increase in smoke-impacted PM_2.5._ For all-cause respiratory visits, we observed a lag-specific OR of just greater than 1 across lag days 0–4, and for asthma visits, we observed an OR of 1.01 (CI: 1.01, 1.01) on lag day 0, and lower ORs through lag 3, with an increase in the OR on lags 4 and 5. For all-cause cardiovascular and MI visits, we observed a lag-specific OR of 1 or slightly greater than 1 across most lag days (figure 2, table S12). Modeling the ORs cumulatively across the lag period shows an upward trajectory in cumulative risk for all outcomes (figure S2, table S13). Results from a quasi-Poisson model examining a lag-specific and cumulative effect of exposure were nearly unchanged (tables S14 and S15).

In secondary exploratory analyses comparing the effect of wildfire smoke versus no wildfire smoke exposure, when stratified by age group (table S6), results were consistent with a range of effects. For all-cause respiratory visits, results for ages 0–18 and ⩾65 showed a decrease in risk or no increase in risk across all lags (table S6). Results for ages 19–64 showed an increase in effect across all lags (ages 19–44) or lags 1–3 (ages 45–64). The results for asthma visits were similar, with generally higher ORs across all age groups. Children ages 5–18 were at increased risk of asthma visits on lags 0–1, with no further lagged effect. For all-cause cardiovascular ED visits, there was evidence of a range of effects across age groups, with a decreased risk for lags 0–2 among ages 65–84, and an increased risk among ages 19–44 for lags 4–6. For MI visits, we again observed a range of effects across age groups. Among ages ⩾65, we observed a decreased risk on lags 0–1 (ages 65–84) or lags 0–3 (ages ⩾85), and an increased risk among ages 19–44 on lags 2–7, and among ages 45–65 on lag 7.

We conducted a sensitivity analysis using a different binary indicator of exposure for our presence versus absence models. The results using the HMS smoke plumes were similar to the results with the primary exposure surface (table S7). Like the primary analysis, the effect estimates for respiratory-related visits were elevated across the lag period, and for asthma-related visits was largest immediately following initial exposure (lag 0–1). Both cardiovascular and MI ED visits had the largest effect several days following initial smoke exposure (lag 4–5 for cardiovascular, and lag 3–7 for MI).

In an additional sensitivity analysis, we aimed to understand the impact of COVID-19 diagnoses on our results from the presence versus absence models. In 2020, we removed all visits with a COVID-19 diagnosis (*N* = 8328), and found our results were nearly unchanged (table S8). We also ran our main binary model excluding visits from 2017 due to differences in patterns of reporting in 2017 compared to 2018–2020 and due to the low number of EDs reporting visit data in 2017 (table S9). Results were consistent with the main presence versus absence model (figure 1; table S4), although reported ORs were slightly larger across all outcomes and lags. We additional reported results stratified by year (table S10). We observe the largest ORs and tightest confidence intervals in 2020, the year with all EDs included in the data. In a final sensitivity analysis, we ran all models using a quasi-Poisson time series analysis and found all results to be nearly unchanged (table S11).

In a final secondary analysis, we sought to visualize the wildfire smoke-health effect relationship within the context of our existing public health messaging. Figure S3 models the exposure-response curve for all-cause respiratory visits on lag day 1 for a 10 *µ*g m^−3^ increase in total PM_2.5_ across the good, moderate, unhealthy for sensitive groups, unhealthy, and very unhealthy categories of the US Environmental Protection Agency’s AQI. For all-cause respiratory visits, there was an approximately linear increase in odds as PM_2.5_ increases. However, there is a large amount of uncertainty in the dose-response in the high end of the PM_2.5_ range. Thus, we only modeled the exposure-response from 0–200 *μ*g m^−3^, as there was not enough exposure data above this threshold to yield meaningful information.

## 4. Discussion

We leveraged a comprehensive dataset to learn how wildfire smoke presence and intensity impact Washington, using syndromic surveillance data covering the entire state. We observed an increase in the odds of all-cause respiratory and asthma visits across the lag period, both comparing wildfire smoke vs non-wildfire smoke days, and for a 10 *μ*g m^−3^ increase in smoke PM_2.5_. We observed an increase in the odds of all-cause cardiovascular and MI visits on lag day 6 and lag days 4–7, respectively, comparing wildfire smoke to non-wildfire smoke days, while our models estimate a protective effect at lag day 0. Additionally, we observed a small increase in both cardiovascular and MI visits when exposure is modeled per 10 *μ*g m^−3^ increase in smoke PM_2.5_.

Our respiratory results are consistent with the estimates reported in the literature, although our results are somewhat more muted. Other studies generally reported increased risk of respiratory impacts over the first 0–2 d following initial wildfire smoke exposure, particularly for asthma, although the magnitude of effect varies, and are larger than what we report in our continuous analysis [15, 19, 35]. Alman *et al* reported results across lag 0–2 that are consistent with an increased risk of asthma ED visits in Colorado using a case-crossover design. They reported an asthma lag 0 OR of 1.04 (95% CI: 1.02, 1.06) for a 5 *μ*g m^−3^ increase in PM_2.5_ [15]. Similarly, Hahn *et al* reported results in Alaska using a case-crossover design across lag days 0–4 that are consistent with an increased risk of asthma ED visits, and with no increase in risk on lag day 5. Their reported same-day asthma OR was 1.13 (95% CI: 1.10, 1.16) for a 10 *μ*g m^−3^ increase in wildfire smoke PM_2.5_ [19], similar to the OR reported by Alman *et al*. They also reported ORs across lag days 0–5 for all respiratory visits, which were increased on lag 0 (OR: 1.04; 95% CI: 1.02, 1.06), and consistent with no increase across lag days 1–5. Stowell *et al* reported a 3 day average OR for asthma and respiratory visits (asthma OR: 1.08 (95% CI: 1.06, 1.11); respiratory OR: 1.02 (95% CI: 1.01, 1.03)) for a 1 *μ*g m^−3^ increase in wildfire smoke PM_2.5_ in Colorado using a case crossover design [35]. These results are much larger than those found by Alman *et al* and Hahn *et al*, as they report per 1 *μ*g m^−3^ increase in wildfire smoke PM_2.5_.

Effect estimates reported in the literature for cardiovascular ED visits are inconsistent. Like our results, Wettstein *et al* reported no increase in the risk of all-cause cardiovascular visits on smoke versus non-smoke days across lag days 0–4 [16]. Additionally, they found evidence for no increased risk of MI visits across all lag days. However, to our knowledge, no studies report a lagged effect of wildfire smoke on cardiovascular visits past 5 lag days. We observed increased risk of all-cause cardiovascular and MI visits several days after initial wildfire smoke exposure in both our presence versus absence and intensity models, suggesting a lagged effect of wildfire smoke on cardiovascular-related ED visits. It is possible that previous studies may have missed this lagged effect by not examining a long enough lag period. In the literature on PM_2.5_ from non-wildfire smoke sources, several studies report a lag of 0–2 d for MI outcomes [36–38]. In our study, we find an increase in MI-related ED visits for a lag of 3–7 d. An exposure to increased PM_2.5_ may initiate a process of plaque rupture which can increase the risk of an MI [39]. However, this process can take a couple of days, explaining the delayed effect in the general PM_2.5_ exposure setting. In a wildfire smoke setting, based on increased wildfire smoke messaging and awareness, individuals may change their behavior by staying inside for the first couple days of an exposure event to reduce their exposure, which may delay MI events by additional day(s) compared to a setting of general PM_2.5_ exposure. Additionally, we observed a strong delayed effect for MI outcomes in 2020 (table S10), suggesting a possible confounding effect of the COVID-19 pandemic when individuals may have been delaying care more than usual [40, 41]. Furthermore, the high prevalence of multi-day smoke events during the study period may contribute to the delayed lag structure. We report lag-specific effects, however, the presence of multi-day smoke events builds cumulative exposure into this analysis. Thus, the effects we observe are likely not solely due to the initial smoke exposure on lag 0.

We aimed to understand the impact of wildfire smoke intensity by modeling the impact of a 10 *μ*g m^−3^ increase in smoke PM_2.5_ on ED visits. The ORs from the lag-specific model are much smaller compared to those from the presence versus absence model, as we used different exposure metrics, and a distributed lag versus a single lag model. We found evidence of increased odds of respiratory and cardiovascular-related ED visits for each 10 *μ*g m^−3^ increase in smoke PM_2.5_, and our results follow similar trends to those reported in our presence versus absence analysis. In a secondary analysis, we examined the cumulative impact of exposure to a 10 *μ*g m^−3^ increase in smoke PM_2.5_ over the full lag period. Few studies report the cumulative impact of wildfire smoke exposure over several days on health outcomes [17, 22]. Rappold *et al* reported the cumulative impact of peat bog smoke versus no smoke across five days, and Yao *et al* reported the cumulative OR of ambulance dispatches over 48 h. Like our study, both studies found evidence of an increase in cumulative outcome-specific risk. These findings suggest a possible need for risk messaging in the several days following initial wildfire smoke exposure.

Our exposure-response curve shows an approximately linear increase in the odds of next day respiratory ED visits per 10 *μ*g m^−3^ increase in PM_2.5_. This result indicates that an increase in PM_2.5_ from 30 to 40 *μ*g m^−3^ may lead to a similar increase in the odds of next day respiratory ED visits as an increase from 100 to 110 *μ*g m^−3^, for example. This finding suggests a possible need for increased risk messaging when air quality is in lower ranges of the AQI (Moderate and Unhealthy for Sensitive Groups) in addition to higher ranges (Unhealthy and above). Above a concentration of 150–200 *μ*g m^−3^, there is a high degree of uncertainty in the true increase in odds due to the relatively few days above this threshold during our study period. Further work is needed to clarify how much the highest intensity of wildfire smoke impacts health outcomes.

Our analysis was bolstered by using syndromic surveillance data. Washington State requires all EDs to report health encounter information. Thus, by the last year of the study period, we were able to capture visits to all EDs in Washington, allowing us to make inference across a large population and geographic area, rather than within one health care system or metropolitan area. Syndromic surveillance data also come with some limitations. The quality of the visit data varied by hospital and may have varied across the reporting period; the data is only as good as what is entered by the hospital. To try to overcome this, we identified cases by using a combination of ICD-10 codes and queries in the chief complaint section. Furthermore, the syndromic surveillance platform in Washington was actively onboarding EDs during the study period, thus there was a different population captured by the data across time. This may have varied differentially by smoke versus non-smoke exposure areas, possibly yielding invalid inference. Thus, we chose to conduct a time-stratified case crossover analysis rather than a Poisson time series analysis, as the case crossover design does not require the population at risk to be known. Additionally, the time-stratified case crossover design compares exposure within each referent window, which is within a single month-year, decreasing the likelihood of bias. However, with the case crossover design, we assume patients do not have readmissions during their referent window. We cannot know this, since these data are visit-based rather than population based. While this is a limitation, we are confident in our results, as they are very similar to those from the Poisson time series analysis, which does not make this assumption. Overall, these data capture ED visits across a large population over four wildfire smoke seasons yielding effect estimates with high certainty relative to other studies.

Our study period included the initial period of the COVID-19 pandemic in June-September 2020. This was also a major wildfire smoke year for Washington. To ensure any effect we might see was not due to the increase in COVID-related ED visits, we ran a sensitivity analysis removing all ED visits with a COVID-19 diagnosis code. We found our effect estimates were nearly unchanged after removing these visits (table S7). We additionally reported results separately by year to better understand differences in EDs reporting by year (table S10). We find that visits from 2020 dominate most of the positive effect we observe, and results from 2017 visits report protective effects, suggesting that the low coverage of EDs reporting visit data may have yielded biased estimates for 2017. We additionally re-ran our primary analysis excluding visits from 2017 due to the low number of EDs reporting that year and to observed differences in outcomes reported (table 1). We observed similar though slightly elevated effect estimates after removing 2017 compared to the primary model with 2017 visits (table S8), suggesting that visits from 2017 are not overly impacting our overall estimates.

We utilized a previously developed exposure surface, population-weighted to the zip code level [25] (Note S1). This surface leverages monitored and modeled PM_2.5_ concentrations to better estimate daily local PM_2.5_ concentrations compared to a model with only monitored PM_2.5_. To assess the sensitivity of our results to the exposure model for the presence versus absence analysis, we used NOAA’s HMS smoke plume daily data as a binary smoke exposure indicator, an exposure metric widely used in epidemiologic applications [16, 19, 42–45]. We found the results from this analysis generally agree with our primary analysis leading to similar conclusions (table S6). However, both our smoke exposure surface and the HMS smoke plumes likely result in some degree of misclassification. For the HMS smoke plume exposure, we only used smoke polygons categorized as ‘Heavy’, which tend to capture all major wildfire smoke plumes, but may miss some of the smoke-impacted days at lower PM_2.5_ concentrations. On the other hand, our wildfire smoke exposure surface may be classifying some days as wildfire smoke-impacted that are not truly smoke-impacted. However, the results from these two methods yielded similar risk estimates (table S6).

## 5. Conclusion

This study adds to the growing body of research supporting evidence of the adverse impacts of wildfire smoke exposure on population health. We reported an increase in all-cause respiratory ED visits, asthma ED visits, and a lagged increase in all-cause cardiovascular and MI-related ED visits using a large statewide dataset over four wildfire smoke seasons in Washington state. We find all-cause respiratory visits are most elevated among ages 19–64, and asthma visits are most elevated among ages 5–64, suggesting that messaging and risk communication need to be targeted toward younger and middle-aged adults.

## Data Availability

The data cannot be made publicly available upon publication because they contain sensitive personal information. The data that support the findings of this study are available upon reasonable request from the authors.
